# Synaptopodin-2: a potential tumor suppressor

**DOI:** 10.1186/s12935-023-03013-6

**Published:** 2023-08-07

**Authors:** Zequn Zheng, Yongfei Song

**Affiliations:** 1 Ningbo Institute of Innovation for Combined Medicine and Engineering, Ningbo Medical Centre Lihuili Hospital, Ningbo University, No. 378 Dongqing Road, Yinzhou District, Ningbo, 315048 Zhejiang People’s Republic of China; 2 School of Laboratory Medicine and Bioengineering, Hangzhou Medical College, Hangzhou, 310012 Zhejiang People’s Republic of China; 3 Medical College, Ningbo University, Ningbo, 315211 Zhejiang People’s Republic of China; 4 Department of Cardiology, Shantou University Medical College, Shantou, 515063 Guangzhou People’s Republic of China

**Keywords:** Synaptopodin 2, YAP1, Mitophagy, Molecular Chaperone-Assisted Selective Autophagy, Single nucleotide polymorphism

## Abstract

Initially identified as an actin-binding protein containing a PSD95-DLG-ZO1 Domain (PZD domain), Synaptopodin 2 (SYNPO2) has long been considered a structural protein ubiquitously expressed in muscular tissues. However, emerging evidence suggests that SYNPO2 performs diverse functions in cancers in addition to its role in microfilament assembly. In most cancers, high SYNPO2 expression is positively correlated with a good prognosis, suggesting its role as a novel tumor suppressor. Abnormal SYNPO2 expression affects autophagy generation, particularly mitophagy induced by low oxidation or viral infection, as well as chaperone-mediated autophagy triggered by microfilament damage. Mechanically, SYNPO2 regulates tumor growth, metastasis, and invasion via activating the PI3K/AKT/mTOR signal and Hippo signaling pathways. Moreover, the subcellular localization, promoter methylation and single nucleotide polymorphism (SNP) of SYNPO2 have been associated with cancer progression and clinical outcomes, highlighting its potential as a prognostic or diagnostic target for this patient population. This review focuses on the role of SYNPO2 in cancer, including its generation, epigenetic modification, subcellular localization, and biological function.

## Introduction

Synaptopodin-2 (SYNPO2), known as Myopodin or Fesselin, was first identified in chicken sand cysts in 1999. It has an isoelectric point (PI) of 9.3 and molecular weights of 79 kDa and 103 kDa [1, 2]. In 2001, Lin et al. documented a 54 kb minimal common deletion region on chromosome 4q25 of the genome associated with invasive prostate cancer [3]. Subsequently, the *SYNPO2* gene, approximately 3.6 Kb, has been identified in mouse skeletal muscles and cardiomyocytes [4].

Recent studies have reported that the SYNPO2 protein acts as a tumor suppressor and is downregulated in most tumor types to enhance tumor development (Table 1). For instance, MYCN inhibits the expression of Dickkopf-1 (DKK1) in neuroblastoma, leading to cell proliferation. This effect is not dependent on activation of the WNT signaling cascade, but is instead due to decreased SYNPO2 expression [5]. A higher ratio of cytoplasmic to nuclear SYNPO2 in patients with hepatocellular carcinoma (HCC) has been linked to an increased likelihood of recurrence, and CaN-induced nuclear-cytoplasmic shuttling of SYNPO2, facilitating the metastasis of HCC by promoting the assembly of peripheral actin bundles [6]. In cervical cancer, the inactivation of SYNPO2 expression promotes tumor development by regulating ER, PI3K/AKT, and EMT pathways [7]. It has been established that in colorectal cancer (CRC), SYNPO2 expression is significantly lower compared to adjacent tissues. Exogenous SYNPO2 can inhibit the occurrence and development of colorectal cancer by upregulating the expression of Proto-Oncogene C-Fos (FOS) and its downstream factors [8]. In prostate cancers, SYNPO2 exhibits dual functions in regulating tumor progression, with some reports showing that its C terminal region inhibits cancer invasion and metastasis while others support the notion that some isoforms of SYNPO2 increase chemokinetic properties and promotes migration [9, 10]. Moreover, low SYNPO2 expression has been detected in bladder cancer [11], breast cancer [12], melanoma [13] and kidney cancer [14].

**Table 1:** Relationship between SYNPO2 expression and cancers

Types of cancer	Treatment	SYNPO2	Signaling pathway	Biological function
Prostate cancer	SYNPO2 inactivation and deletion	Low	NA	Clinical relapse [3, 67]
	Overexpression	Up	Interaction with zyxin	Inhibiting tumor growth and metastasis [9]
			NA	To promote lamellipodia formation and cell migration [10, 18]
	siSYNPO2	Low	NA	Inhibiting invasion and motility [65, 66]
	Phosphorylation of SYNPO2	NA	NA	Cell growth and motility [23]
Neuroblastoma	DKK1 induce SYNPO2 expression	Up	NA	Growth suppressive effect [5]
Hepatocellular carcinoma	Cytoplasmic translocation	Low	CaN/SYNPO2/F-actin axis	Metastasis [6]
Cervical cancer	miRNAs downregulated SYNPO2	Low	ER;PI3K/AKT;EMT signaling pathway	Inhibiting DNA damage and cell cycle [7]
Colorectal cancer	Overexpression	Up	YAP-KLF5 axis [47]; FOS [8]	Inhibiting proliferation and migration [47]; Inhibiting cell proliferation [8]
	Down-regulation of SYNPO2	Low	ER, PI3K/AKT, EMT signaling pathway	Inhibiting DNA damage and cell cycle [7]
	Azacitidine	Low	NA	Associated with tumor stage and survival [58]
Melanoma	Methylation	Low	NA	Recurrence [13]
	Endogenous	Low	TME	Anti-PD-1 therapy [56]
Kidney cancer	Methylation	Low	NA	Distant metastasis and survival [14]
Triple negative breast cancer	Overexpression [45, 51]; vitamin C [46]	Up	YAP/TAZ [45]; PI3K/Akt/mTOR pathway [51]; YAP1 [46]	Inhibiting stem cell-like properties, clinical stage, survival, lymph node metastasis [45, 46, 51];
	LncRNA	Low	NA	Inhibiting relapse-free survival of TNBC [12]
Bladder cancer	Methylation	Low	NA	Associated with tumor stage and tumor grade [59, 60]
	Endogenous	Low	NA	Inhibiting survival and an increased recurrence rate [11]

Despite the growing number of studies on SYNPO2, a review of its tumor suppressor mechanisms has not been published. This paper details the characteristics of SYNPO2 and explains the role of SYNPO2 in tumorigenesis and development, including epigenetic modification, subcellular localization, signaling pathway, autophagy, and chaperone-assisted autophagy. In addition, the potential and value of SYNPO2 single nucleotide polymorphisms (SNPs) and promoter region methylation as tumor diagnostic markers are explored.

## About SYNPO2 gene

### Genomic features of *SYNPO2*

The *SYNPO2* gene is highly conserved across multiple species, including mice, humans, rats, chickens, and frogs. The human *SYNPO2* gene spans 273,737 base pairs, comprises seven exons, and is located on chromosome 4q26 (NC_000004.12), with 74,692 reported single-nucleotide polymorphism sites (http://www.ncbi.nlm.nih.gov/snp). The human mRNA of SYNPO2 is 7282 nucleotides in length and is composed of five exons, with the coding sequence originating from exons 1 to 5, spanning from 184 to 3969 bp (NCBI NM_133477, http://www.ncbi.nlm.nih.gov).

### Transcripts of *SYNPO2* gene

SYNPO2 transcripts have been identified in various tissues, including murine and human muscle, heart tissue, bovine breast tissue, rabbit stomach, and chicken sac. It is now understood that the gene generates seven transcripts through transcription and alternative splicing, corresponding to seven SYNPO2 protein (a–g) isoforms. The methylation levels of CpG islands near the promoter region regulate the transcription of *SYNPO2* gene. For example, increased methylation of the SYNPO2 promoter region reduced transcription in the muscles of women with polycystic ovary syndrome [15].

Interestingly, the *SYNPO2* gene also produces a long non-coding RNA, called SYNPO2 intron sense overlapping lncRNA (SYISL), via alternative splicing. Mridula P. Menon reported that SYISL could promote C2C12 cells migration and proliferation by activating downstream effector proteins of the WNT signaling pathway, including β-catenin and PKC. It sponged miR-23a-3p, miR-103-3p, or miR-205-5p to upregulate muscle atrophy genes, including Forkhead box protein O3a (FoxO3a), muscle ring finger 1 (MuRF1), and muscle atrophy-related F-box (Atrogin-1), and stimulate the generation of muscle atrophy [16]. Moreover, SYISL could inhibit muscle development by recruiting the Enhancer of Zeste Homolog 2 (EZH2) protein and inducing H3K27 trimethylation in the promoter of p21 and muscle-associated genes, including Myogenic Regulation Factor (MyoG), Muscle Creatine Kinase (MCK), and Myosin Heavy Chain 4 (Myh4) [17].

### About SYNPO2 protein

#### SYNPO2 isoforms and structure

Human SYNPO2 proteins belong to the synaptopodin protein family. The overall homology identity between myopodin and synaptic proteins is 47.7%, with the highest homology observed at the C terminus of the protein. As mentioned above, seven isoforms of SYNPO2 protein have been identified, with isoform a being the longest and having a molecular weight of about 110 kDa. The homology between human and mouse SYNPO2 is 87.5% in this isoform. The different isoforms of SYNPO2 have varying biological functions in actin bundle formation. For example, overexpression of isoform a or isoform d induces the formation of long, well-ordered actin bundles, whereas isoform g generates a randomly-ordered, thick, and irregular actin bundle network at the center of the cell body [18].

**Fig. 1:**
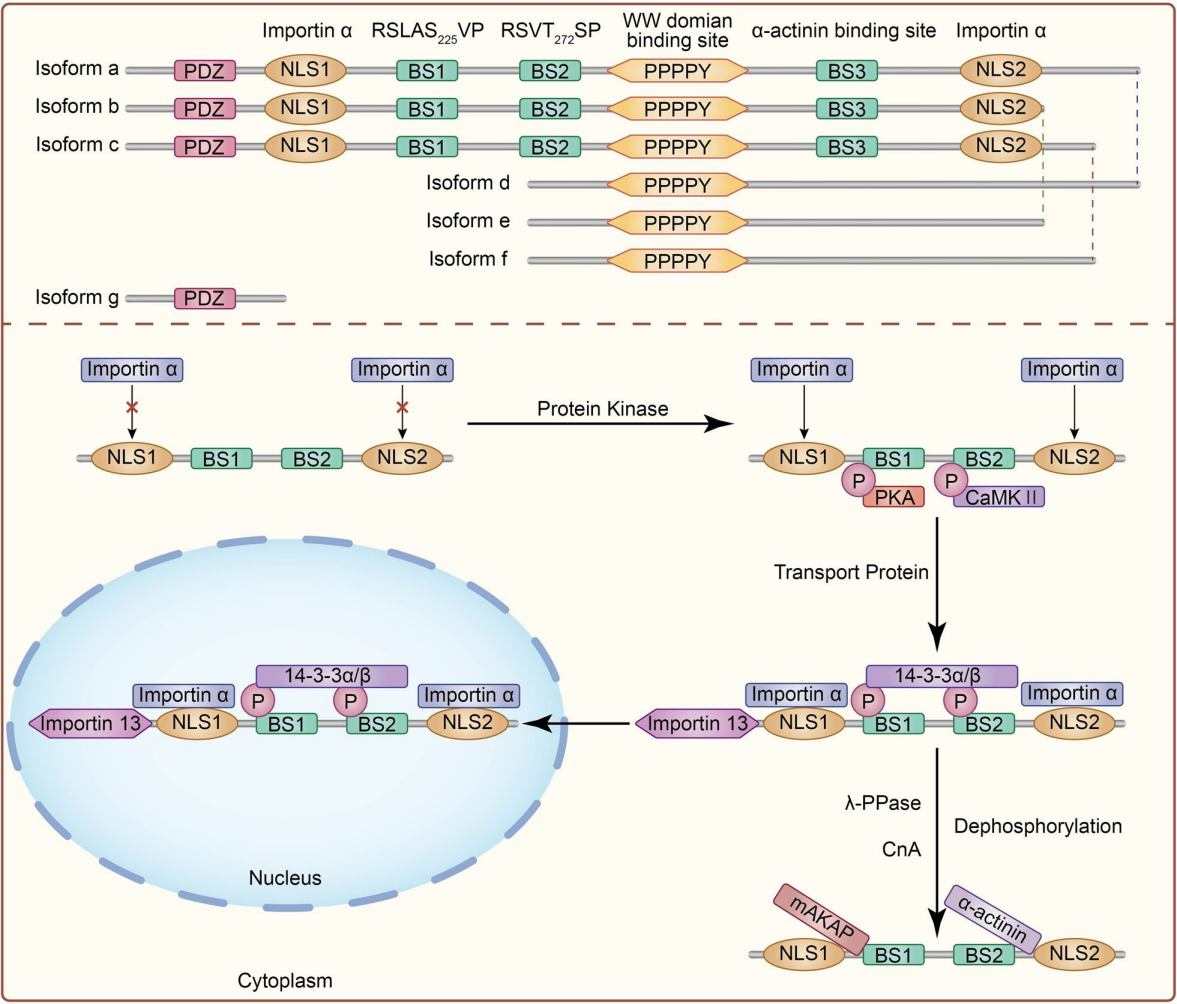
The molecular mechanism of SYNPO2 nuclear-cytoplasmic shuttling. The upper part depicts six different isoforms of SYNPO2 including their motifs, domains and signaling sequences, while the lower part shows the process of nuclear-cytoplasmic shuttling of SYNPO2, which typically involves four stages: (1) the 14-3-3 beta binding sites (BS) on SYNPO2 are phosphorylated by PKA or CaMKII; (2) after phosphorylation of SYNPO2, Importin α and β interact with the nuclear localization sequence of SYNPO2; (3) importin α and β lead SYNPO2 shuttling from cytoplasm to nucleus. (4) If many phosphatases such as λ-PPase and CnA are activated, dephosphorylation of SYNPO2 induces binding with mAKAP and α-actinin to keep its retention in the cytoplasm

SYNPO2 protein has long been considered a structural protein, which colocalizes with actin near the Z axis. A variant of the SYNPO2 protein called Fesselin was isolated from chicken gizzard muscle that could still polymerize actin even in the presence of actin inhibitors [19]. It is widely thought that the PDZ domain, located at F6-S88 in SYNPO2 protein, may contribute to binding to other proteins and lipids on the cell membrane [20]. Several hydrophobic PXXP motifs were identified as potential sites for binding to SH3 domain-containing proteins. In contrast to the synaptopodin protein, SYNPO2 contains only one PPXY motif in humans and mice. The PPXY motif is also essential in mediating protein–protein interactions, especially in the WW domain. For example, PPPY can bind WW motifs of BAG3 to mediate the occurrence of chaperone-assisted autophagy [21, 22] (Fig. 1).

#### Protein modification in SYNPO2

Protein modification can impact various cellular processes, including enzyme activity, protein turnover and localization, protein–protein interactions, modulation of signaling cascades, DNA repair, and cell division. Several phosphorylation sites in SYNPO2 protein have been identified, including S902, S906, S604, S638, S226, S611, and T626. Phosphorylation modification alters the biological function of SYNPO2. For example, the ILK-dependent kinase activated by Integrins α7 can phosphorylate SYNPO2 to inhibit prostate cancer proliferation and migration both in vivo and in vitro. This phenomenon may be associated with the phosphorylation-mediated transformation of SYNPO2 between nuclei and cytoplasm [23]. Of significance, phosphorylation mediates the cellular nuclear localization of the SYNPO2 protein, which will be discussed in the subsequent sections.

#### Subcellular localization of SYNPO2

It has been shown that the inappropriate subcellular localization of proteins affects tumorigenesis and development. Key factors such as CCCTC-Binding Factor Like (CTCFL) can exert pro-apoptotic effects in the nucleus but suppress apoptosis in the cytoplasm [24]. It has been established that different nuclear localization sequences regulate this process. The SYNPO2 protein harbors two conserved classical nuclear localization signals (NLSs) in humans and mice. These NLSs are located at the N-terminus (amino acid sequence: 58KKRRRRARK66) and the C-terminus (amino acid sequence: 612KTSKKKGKK620) of the protein, respectively [25]. In prostate cancer PC3 cells, GFP-tagged SYNPO2 truncation M56-P432 is strongly localized in the nucleus, while GFP-tagged SYNPO2 truncation M219-E758 is primarily distributed in the cytoplasm. These findings suggest that N-terminal NLS is more important than the other NLS in the subcellular distribution of SYNPO2. Besides, SYNPO2 protein also contains one nuclear export signal (NES) region P3-L24. The absence of NES increased SYNPO2 nuclear localization to inhibit the invasion of prostate cancer cells.

Most studies have shown that low SYNPO2 expression in the nucleus is associated with tumorigenesis and cancer development. The nuclear translocation of SYNPO2 is mediated by interaction with 14-3-3β and the involvement of importin family proteins [26]. Phosphorylation of SYNPO2 at S225 and T272 sites by PKA and CamKII, respectively, is critical for its nuclear import, mediated by 14-3-3β binding, and lambda-PPase and CnA mediated dephosphorylation abrogate the activity of 14-3-3β [27]. The A-Kinase Anchoring Protein 6 (mAKAP) inhibits SYNPO2 nuclear importation, while α-actinin overexpression displays a z-disc colocalization with SYNPO2 by competitive interaction with 14-3-3β. Finally, Importin α binds to the NLSs of SYNPO2 proteins to promote its nuclear import, and Importin β plays a crucial role in traversing the nuclear pore complex [28] (Fig. 1).

Compared to high SYNPO2 expression in the nucleus, cytoplasmic SYNPO2 expression was significantly upregulated in cancer patients with high recurrence. Therefore, it is widely thought that nuclear localization of SYNPO2 can inhibit tumor development while high SYNPO2 expression in the cytoplasm accelerates tumor progression.

## SYNPO2 affects autophagy in cancer

Autophagy, encompassing macroautophagy, microautophagy, and molecular chaperone-assisted selective autophagy (CASA), plays a crucial role in cancer by activating autophagy-related (ATG) proteins [29]. One significant example is the aberrant expression of the STAT3 protein, which exerts control over autophagy by regulating the transcription of various autophagy-related genes, including members of the BCL2 family, BECN1, PIK3C3, PIK3R1, BNIP3, as well as microRNAs that target autophagy modulators [30]. This regulatory mechanism is closely associated with the development and progression of cancer. Emerging evidence suggests that SYNPO2 participates in selective autophagy, particularly mitophagy and chaperone-assisted selective autophagy.

### SYNPO2 in mitophagy

**Fig. 2:**
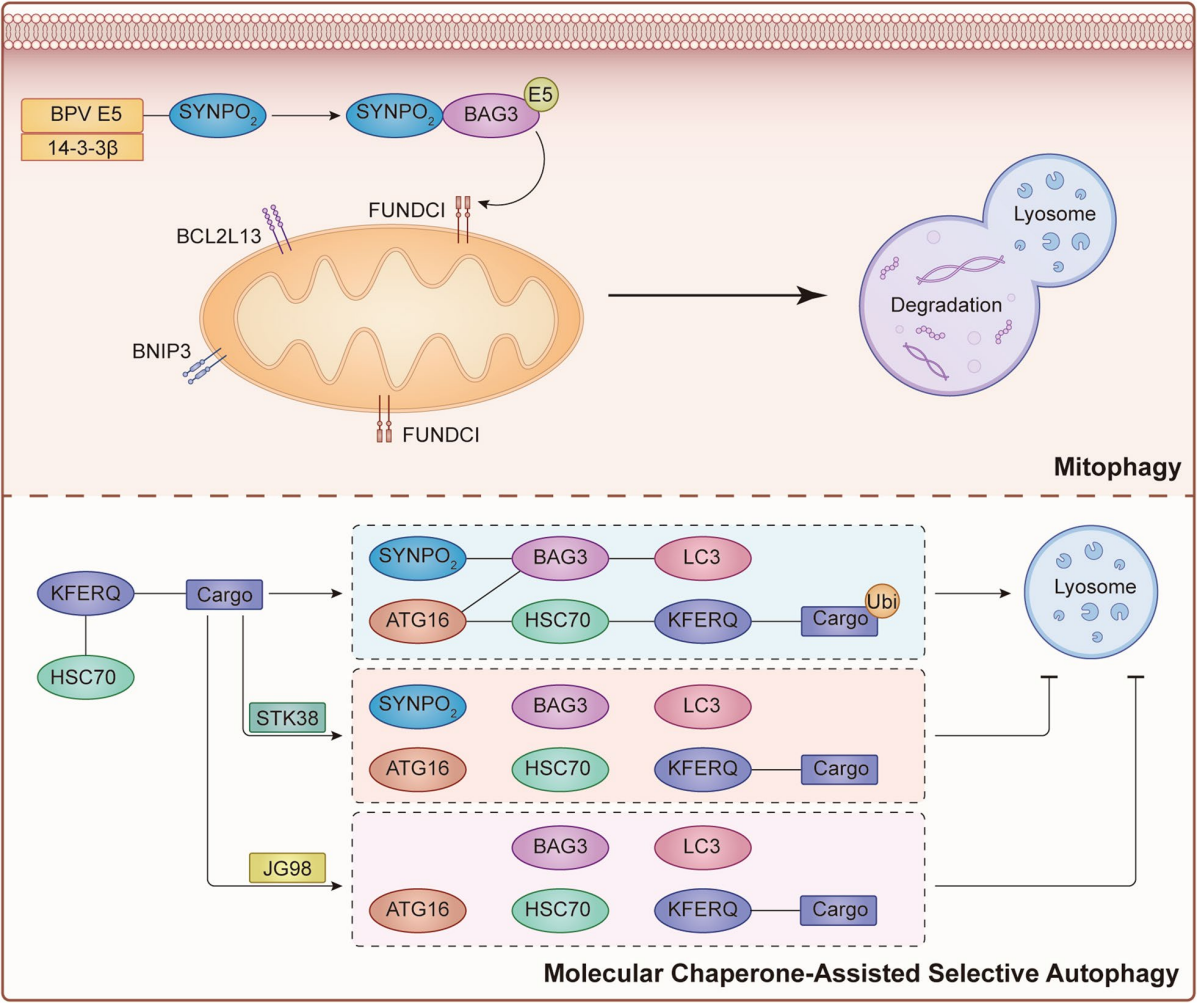
SYNPO2 involve in the process of autophagy. The upper part shows that SYNPO2 interacts with 14-3-3β, BPV-E5, and BAG3, increasing their binding with receptors on the mitochondria and accelerating the accumulation of this complex on the membrane of the mitochondria, leading to the generation of mitophagy. The lower part depicts that SYNPO2 expression facilitates the recruitment of CASA-associated proteins to drive cargo degradation for lysosome-dependent degradation after HSC70 binds with the KFERQ motif. The process is inhibited by blocking the interaction between SYNPO2 and BAG3 or by a lack of SYNPO2, corresponding to overactivation of STK38 and JG98 treatment, respectively

Mitophagy is a form of selective autophagy that involves the engulfment and degradation of dysfunctional mitochondria by lysosomes. It was first described by Lemasters and Priault in 2005 [31, 32]. It is now understood that mitophagy receptors accumulate on the outer mitochondrial membrane and are beneficial to binding to mammalian ATG8 proteins via LC3-interacting regions (LIRs). Three main types of receptors, including BCL2 Interacting Protein 3 (BNIP3/NIX), FUN14 Domain Containing 1 (FUNDC1), and BCL2 Like 13 (BCL2L13), are activated under hypoxia or mitochondrial depolarization [33]. In bovine papillomavirus (BPV)-infected urinary tract cancer cells, a complex consisting of Hsp7, CHIP cargo, SYNPO2, ERAS, LC3, p62, BNIP3, and BNIP3L/Nix, was identified [34]. SYNPO2, Bag3, and BPV E5 oncoproteins colocalize with perinuclear sites, and the interaction between SYNPO2 and 14-3-3β facilitates the retrograde transportation of BPV E5 proteins along microtubules to perinuclear regions characterized by a high level of autophagic flux [35]. Similarly, Roperto S reported the interaction between FUNDC1 and various proteins, such as LC3 (a marker for mitophagosome generation) and co-chaperones Hsc70 and Bag3. Bag3, along with SYNPO2 protein, is believed to have a role in the mitophagosome formation and may take part in the degradation of CHIP-ubiquitinated cargoes in collaboration with molecular chaperones [36]. These findings suggest that SYNPO2 contributes to mitophagosome formation and CHIP-ubiquitinated cargo degradation (Fig. 2).

### SYNPO2 in molecular chaperone-assisted selective autophagy

Molecular chaperone-assisted selective autophagy is a highly selective process that involves the recognition of specific pentapeptide motifs by Hsc70. These motifs enable Hsc70 to deliver targeted proteins to lysosomes for degradation via the receptor protein LAMP2A [37]. when cells experience stress, SYNPO2 expression is upregulated and CASA is activated [38]. The PDZ motif present in the SYNPO2 protein can bind to autophagy-associated proteins, namely VSP16, VSP18, and ATG16L1, thereby affecting the occurrence of CASA under stress.

It has been established that in most cancer cell lines, CASA activity is significantly higher compared to normal control cells. The CASA complex, including Hsc70, HSPB80, BAG3, and BNIP ligase, is recruited in response to filamin damage, and the binding of BAG3 and SYNPO2 promotes the formation of autophagy. Another study suggested that JG98 could inhibit the formation of the Hsc70-BAG3 complex by reducing SYNPO2 expression [39]. Similarly, removing SYNPO2 homologs, SYNPO and BAG3, suppresses autophagosome membrane elongation [40]. Furthermore, STK38, a critical factor in the Hippo signaling pathway, can inhibit CASA by blocking the interaction between SYNPO2 and BAG3 proteins, leading to the degradation of SYNPO2 proteins [41] (Fig. 2). However, the reasons behind the upregulation of CASA activity and the abnormal down regulation of SYNPO2 protein expression in cancer remain unclear. Indeed, additional research is required to elucidate the molecular mechanisms underlying the atypical activation of CASA following SYNPO2 inhibition in tumors.

## SYNPO2 regulates key signaling pathways in cancers

The dysregulation of key signaling pathways is an important hallmark of tumor initiation and progression, and it represents significant targets for tumor diagnosis, prognosis, and treatment. Currently, research on SYNPO2 primarily focuses on the Hippo signaling pathway and the PI3K/AKT signaling pathway. These two pathways are crucial in tumor proliferation, migration, invasion, and drug resistance. Therefore, we believe that the anticancer effect of SYNPO2 is closely intertwined with these pathways.

### SYNPO2 in the Hippo signaling pathway

The Hippo signaling pathway was first discovered in Drosophila to primarily regulate tissue and organ development [42]. Increasing evidence suggests that dysregulation of the Hippo pathway, plays a crucial role in tumorigenesis and cancer development. In mammals, the Hippo homolog gene MST1 (mammalian STE20-like protein kinase 1, STK 4) and MST2 (STK 3) have been identified [43]. Upon stimulation by upstream signals, the MST 1/2 kinase undergoes autophosphorylation and activates downstream kinases such as LATS1 and LATS2, facilitating the phosphorylation of Yes-Associated Protein (YAP) and WW Domain Containing Transcription Regulator 1 (TAZ), leading to their cytoplasm retention. However, dephosphorylated YAP/TAZ are transported into the nucleus to activate the TEAD family proteins-mediated transcription system in tumorigenesis, ultimately controlling cell proliferation, differentiation, and apoptosis [44].

Recent literature has reported that SYNPO2 stabilizes the LATS2 protein, promoting the phosphorylation of YAP1 at S127 and leading to the retention of YAP and TAZ in the cytoplasm, restraining the TEAD transcription activity and downstream gene expression such as SRY-Box Transcription Factor 2(SOX2), NONAG and POU Class 5 Homeobox 1(POU5F1/OCT4), to inhibit the metastasis of triple-negative breast cancer [45]. Vitamin C increases the expression of synaptopodin 2 while decreasing the expression of genes in the Hippo pathway, such as YAP1 [46]. Additionally, as a transcription cofactor, YAP1 can induce the expression of Kruppel-like factor 5 (KLF5), thus promoting the expression of HIF-1a and resisting hypoxia. In the colorectal cancer cell lines LOVO and HT29, the overexpression of SYNPO2 under hypoxic conditions was found to result in the suppression of YAP1, KLF5, and HIF-1α expression [47]. Consequently, it is highly conceivable that elevated SYNPO2 expression inhibits cell migration, and the EMT signaling pathway, and induces apoptosis.

**Fig. 3:**
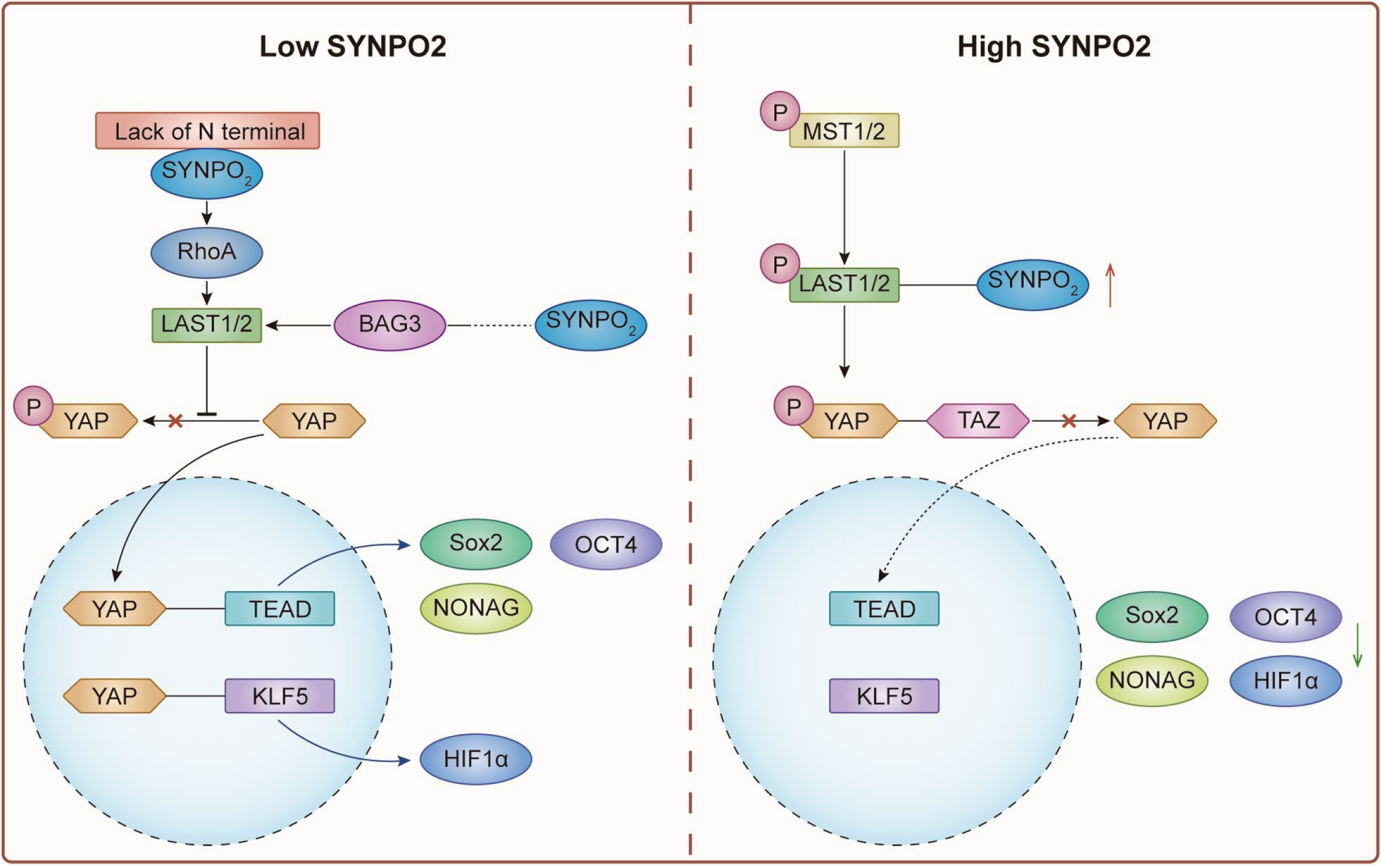
The role of SYNPO2 in Hippo pathway signaling. The left part shows that low SYNPO2 or abnormal SYNPO2 expression increases the nuclear importation of YAP, leading to the activation of transcriptional factors such as TEAD and KLF5, which promotes cancer stemness and resistance to oxidative stress. The right part indicates that high SYNPO2 expression promotes the phosphorylation of YAP, resulting in the cytoplasmic localization of YAP and the inactivation of its co-transcription

SYNPO2 plays a crucial role in regulating the activity of YAP1 through cross-talk with the RHO-ROCK signaling pathway. Studies have reported that RhoA can inhibit the activity of LATS1 and promote the nuclear localization of YAP1. A new SYNPO2 transcript lacking the N terminus was found to promote prostate cancer cell invasion by activating RHO-ROCK signaling, indicating that abnormal SYNPO2 can initiate YAP1 nuclear translocation as well as transcriptional activity and promote cancer progression [48] (Fig. 3). Moreover, SYNPO2 overexpression increases the phosphorylation of YAP1 by inducing activation of LATS1 and supports BAG3 to assemble autophagosomes.

### SYNPO2 in the PI3K/AKT signaling pathway

The PI3K/Akt/mTOR signaling pathway regulates numerous cellular biological functions, such as cell growth, metastasis, survival, and metabolism [49]. For instance, CircIL4R activates the PI3K/AKT signaling pathway to promotes proliferation and metastasis in colorectal cancer [50]. There is a rich literature available substantiating that SYNPO2 expression is negatively associated with BC lymph node metastasis and stage. Knockdown of SYNPO2 enhances phosphorylation of AKT and mTOR to promote migration and invasion in various breast tumor cell lines, including MCF-7, MDA-MB-231, BT-549, and MDA-MB-468 [51]. Indeed, further investigation is needed to fully elucidate the mechanism by which SYNPO2 knockdown induces the activation of the PI3K/AKT signaling pathway.

## The role of SYNPO2 in immune response in different cancers

SNPs, the most common human heritable variation, can directly impact protein structure or expression levels, resulting in specific biological traits in disease inheritance [52]. For instance, SNPs in TP53 and EGFR have been shown to affect their functions in cancer [53], suggesting they are useful for disease prognosis evaluation and genotype analysis. Recent studies have linked SYNPO2 gene SNPs to the progression of epithelial ovarian cancer (EOC) [54] and the risk of familial colorectal cancer.

An association between SYNPO2 SNPs and immunity has been found, with SYNPO2 rs1038770 associated with total IgE levels and mutations detected in patients with nephrotic syndrome [55]. A six-gene panel, including SYNPO2, has been identified for predicting the response of melanoma to anti-PD-1 therapy [56]. Decreased SYNPO2 mRNA levels promote the development of HIV-associated lung cancer [57]. While the specific role of SYNPO2 in tumor immunity requires further investigation, existing evidence suggests that SYNPO2 indeed plays a significant role in this context. Thus, exploring the involvement of SYNPO2 in tumor immunity is considered a promising avenue for future research on SYNPO2 molecules.

## SYNPO2 methylation in cancer diagnosis and prognosis

It is now understood that the expression of SYNPO2 is controlled by a “switch” mechanism, where promoter methylation leads to suppression of SYNPO2 transcription, while demethylation activates its expression. Methylation occurring in the promoter region of SYNPO2 has been shown to impact its biological function. In this regard, genomic methylation levels of SYNPO2 in colon cancer were found to be negatively correlated with its protein expression, and low SYNPO2 expression exhibited a positive correlation with advanced stage and poor survival [58]. In a cohort study involving 88 kidney tumor samples, 50 cases (56.8%) revealed an association between the methylation status of the SYNPO2 and the Memorial Sloan-Kettering Cancer Center (MSKCC) risk score and distant metastasis [14]. In metastatic melanomas, a significant increase in SYNPO2 promoter methylation level was observed compared to non-metastatic melanoma, and SYNPO2 promoter hypermethylation was highly correlated with poor prognosis [13]. Similarly, in a separate cohort study involving bladder cancer, around 68.7% of *SYNPO2* genes in 466 tumor samples showed hypermethylation, and the methylation levels of *SYNPO2* were significantly associated with tumor stage and grade [59]. Moreover, *SYNPO2* gene methylation was identified in 164 urine specimens from bladder cancer patients, with a specificity of 79.8% and an accuracy of 75.3% compared to controls [60]. Therefore, evaluating SYNPO2 promoter methylation and its expression level could be useful as diagnostic and prognostic cancer predictors.

## Conclusion and future

Based on current studies, SYNPO2 has emerged as more than just a structural protein, playing a significant role in autophagy by interaction with BAG3. This biological function is similar to the GAPDH protein, which acts as a chaperone and interacts with microtubules [61]. Hence, SYNPO2 positively affects cancer, potentially inhibiting apoptosis of cancer cells and maintaining intracellular metabolism. Despite growing research in the field, the molecular mechanisms of SYNPO2 in the process of tumor cell autophagy require further investigation.

High methylation of the SYNPO2 promoter region and low expression of its protein can be used alone or in combination with other factors for tumor diagnosis and risk assessment. On the other hand, low SYNPO2 expression and mutation can activate signaling pathways such as the PI3K/AKT/mTOR and the LATS2/YAP/TAZ pathways, promoting cancer development. Small molecule drugs targeting the Hippo signaling pathways and PI3K/AKT/mTOR have shown promise in clinical trials and offer exciting possibilities for targeted cancer therapy [62, 63]. Although the challenge of drug resistance persists, SYNPO2 could be a valuable candidate for drug development as an upstream regulator of these pathways. Therefore, Screening small molecule activators of SYNPO2 via repurposing of drugs represents a promising strategy for treating tumors [64]. Indeed, further research is needed to fully explore the potential of SYNPO2 as a novel target for cancer treatment.

## Data Availability

Not applicable.

## Abbreviations

SYNPO2: Synaptopodin-2
PDZ domain: PSD95-DLG-ZO1 domain
SNPs: Single-nucleotide polymorphisms
NLS: Nuclear localization signal
NES: Nuclear export signal
RHOA: Ras Homolog Family Member A
BNIP3: BCL2 Interacting Protein 3
FUNDC1: FUN14 Domain Containing 1
YAP1: Yes1 Associated Transcriptional Regulator
TAZ: WW Domain Containing Transcription Regulator 1
LATS2: Large Tumor Suppressor Kinase 2
LATS1: Large Tumor Suppressor Kinase 1
CASA: Molecular chaperone-assisted selective autophagy
BAG3: BAG Cochaperone 3
BC: Breast cancer
EOC: Epithelial ovarian cancer

## References

[1] Leinweber BD, Fredricksen RS, Hoffman DR, Chalovich JM (1999). Fesselin: a novel synaptopodin-like actin binding protein from muscle tissue. J Muscle Res Cell Motil.

[2] Kingsbury NL, Renegar RH, Chalovich JM (2013). Avian synaptopodin 2 (fesselin) stabilizes myosin filaments and actomyosin in the presence of ATP. Biochemistry.

[3] Lin F, Yu YP, Woods J, Cieply K, Gooding B, Finkelstein P, Dhir R, Krill D, Becich MJ, Michalopoulos G (2001). Myopodin, a synaptopodin homologue, is frequently deleted in invasive prostate cancers. Am J Pathol.

[4] Weins A, Schwarz K, Faul C, Barisoni L, Linke WA, Mundel P (2001). Differentiation- and stress-dependent nuclear cytoplasmic redistribution of myopodin, a novel actin-bundling protein. J Cell Biol.

[5] Koppen A, Ait-Aissa R, Hopman S, Koster J, Haneveld F, Versteeg R, Valentijn LJ (2007). Dickkopf-1 is down-regulated by MYCN and inhibits neuroblastoma cell proliferation. Cancer Lett.

[6] Gao J, Zhang HP, Sun YH, Guo WZ, Li J, Tang HW, Guo DF, Zhang JK, Shi XY, Yu DS (2020). Synaptopodin-2 promotes hepatocellular carcinoma metastasis via calcineurin-induced nuclear-cytoplasmic translocation. Cancer Lett.

[7] Hossain MS, Quadery TM, Islam MN, Islam MS, Afif IK, Singha RA, Fariha A, Al Reza H, Bahadur NM, Rahaman MM (2021). MicroRNAs expression analysis shows key affirmation of synaptopodin-2 as a novel prognostic and therapeutic biomarker for colorectal and cervical cancers. Heliyon.

[8] Wang L, Sun Y, Jiang M, Zhang S, Wolfl S (2009). FOS proliferating network construction in early colorectal cancer (CRC) based on integrative significant function cluster and inferring analysis. Cancer Invest.

[9] Yu YP, Luo JH (2006). Myopodin-mediated suppression of prostate cancer cell migration involves interaction with zyxin. Cancer Res.

[10] Kai F, Fawcett JP, Duncan R (2015). Synaptopodin-2 induces assembly of peripheral actin bundles and immature focal adhesions to promote lamellipodia formation and prostate cancer cell migration. Oncotarget.

[11] Sanchez-Carbayo M, Schwarz K, Charytonowicz E, Cordon-Cardo C, Mundel P (2003). Tumor suppressor role for myopodin in bladder cancer: loss of nuclear expression of myopodin is cell-cycle dependent and predicts clinical outcome. Oncogene.

[12] Tian T, Gong Z, Wang M, Hao R, Lin S, Liu K, Guan F, Xu P, Deng Y, Song D (2018). Identification of long non-coding RNA signatures in triple-negative breast cancer. Cancer Cell Int.

[13] Gao L, van den Hurk K, Nsengimana J, Laye JP, van den Oord JJ, Beck S, Gruis NA, Zoutman WH, van Engeland M, Newton-Bishop JA (2015). Prognostic significance of promoter hypermethylation and diminished gene expression of SYNPO2 in melanoma. J Invest Dermatol.

[14] Pompas-Veganzones N, Sandonis V, Perez-Lanzac A, Beltran M, Beardo P, Juarez A, Vazquez F, Cozar JM, Alvarez-Ossorio JL, Sanchez-Carbayo M (2016). Myopodin methylation is a prognostic biomarker and predicts antiangiogenic response in advanced kidney cancer. Tumour Biol.

[15] Nilsson E, Benrick A, Kokosar M, Krook A, Lindgren E, Kallman T, Martis MM, Hojlund K, Ling C, Stener-Victorin E (2018). Transcriptional and epigenetic changes influencing skeletal muscle metabolism in women with polycystic ovary syndrome. J Clin Endocrinol Metab.

[16] Jin J, Du M, Wang J, Guo Y, Zhang J, Zuo H, Hou Y, Wang S, Lv W, Bai W (2022). Conservative analysis of synaptopodin-2 intron sense-overlapping lncRNA reveals its novel function in promoting muscle atrophy. J Cachexia Sarcopenia Muscle.

[17] Jin JJ, Lv W, Xia P, Xu ZY, Zheng AD, Wang XJ, Wang SS, Zeng R, Luo HM, Li GL (2018). Long noncoding RNA SYISL regulates myogenesis by interacting with polycomb repressive complex 2. Proc Natl Acad Sci USA.

[18] Kai F, Duncan R (2013). Prostate cancer cell migration induced by myopodin isoforms is associated with formation of morphologically and biochemically distinct actin networks. FASEB J.

[19] Khaymina SS, Kenney JM, Schroeter MM, Chalovich JM (2007). Fesselin is a natively unfolded protein. J Proteome Res.

[20] Linnemann A, van der Ven PF, Vakeel P, Albinus B, Simonis D, Bendas G, Schenk JA, Micheel B, Kley RA, Furst DO (2010). The sarcomeric Z-disc component myopodin is a multiadapter protein that interacts with filamin and alpha-actinin. Eur J Cell Biol.

[21] Ulbricht A, Eppler FJ, Tapia VE, van der Ven PF, Hampe N, Hersch N, Vakeel P, Stadel D, Haas A, Saftig P (2013). Cellular mechanotransduction relies on tension-induced and chaperone-assisted autophagy. Curr Biol.

[22] Lohanadan K, Molt S, Dierck F, van der Ven P, Frey N, Hohfeld J, Furst DO (2021). Isoform-specific functions of synaptopodin-2 variants in cytoskeleton stabilization and autophagy regulation in muscle under mechanical stress. Exp Cell Res.

[23] Yu YP, Luo JH (2011). Phosphorylation and interaction of myopodin by integrin-link kinase lead to suppression of cell growth and motility in prostate cancer cells. Oncogene.

[24] Zhang Y, Fang M, Song Y, Ren J, Fang J, Wang X (2017). Brother of regulator of imprinted sites (BORIS) suppresses apoptosis in colorectal cancer. Sci Rep.

[25] De Ganck A, Hubert T, Van Impe K, Geelen D, Vandekerckhove J, De Corte V, Gettemans J (2005). A monopartite nuclear localization sequence regulates nuclear targeting of the actin binding protein myopodin. FEBS Lett.

[26] Faul C, Huttelmaier S, Oh J, Hachet V, Singer RH, Mundel P (2005). Promotion of importin alpha-mediated nuclear import by the phosphorylation-dependent binding of cargo protein to 14-3-3. J Cell Biol.

[27] Faul C, Dhume A, Schecter AD, Mundel P (2007). Protein kinase A, Ca2+/calmodulin-dependent kinase II, and calcineurin regulate the intracellular trafficking of myopodin between the Z-disc and the nucleus of cardiac myocytes. Mol Cell Biol.

[28] Liang J, Ke G, You W, Peng Z, Lan J, Kalesse M, Tartakoff AM, Kaplan F, Tao T (2008). Interaction between importin 13 and myopodin suggests a nuclear import pathway for myopodin. Mol Cell Biochem.

[29] Li X, He S, Ma B (2020). Autophagy and autophagy-related proteins in cancer. Mol Cancer.

[30] Garg M, Shanmugam MK, Bhardwaj V, Goel A, Gupta R, Sharma A, Baligar P, Kumar AP, Goh BC, Wang L (2020). The pleiotropic role of transcription factor STAT3 in oncogenesis and its targeting through natural products for cancer prevention and therapy. Med Res Rev.

[31] Priault M, Salin B, Schaeffer J, Vallette FM, di Rago JP, Martinou JC (2005). Impairing the bioenergetic status and the biogenesis of mitochondria triggers mitophagy in yeast. Cell Death Differ.

[32] Lemasters JJ (2005). Selective mitochondrial autophagy, or mitophagy, as a targeted defense against oxidative stress, mitochondrial dysfunction, and aging. Rejuvenation Res.

[33] Panigrahi DP, Praharaj PP, Bhol CS, Mahapatra KK, Patra S, Behera BP, Mishra SR, Bhutia SK (2020). The emerging, multifaceted role of mitophagy in cancer and cancer therapeutics. Semin Cancer Biol.

[34] Roperto S, De Falco F, Perillo A, Catoi C, Roperto F (2019). Mitophagy mediated by BNIP3 and BNIP3L/NIX in urothelial cells of the urinary bladder of cattle harbouring bovine papillomavirus infection. Vet Microbiol.

[35] Roperto S, Russo V, De Falco F, Urraro C, Maiolino P, Del PF, Roperto F (2019). Bovine papillomavirus E5 oncoprotein expression and its association with an interactor network in aggresome-autophagy pathway. Vet Microbiol.

[36] Roperto S, Russo V, De Falco F, Rosati A, Catoi C, Roperto F (2019). FUNDC1-mediated mitophagy in bovine papillomavirus-infected urothelial cells. Vet Microbiol.

[37] Ulbricht A, Gehlert S, Leciejewski B, Schiffer T, Bloch W, Hohfeld J (2015). Induction and adaptation of chaperone-assisted selective autophagy CASA in response to resistance exercise in human skeletal muscle. Autophagy.

[38] Salim C, Muders H, Jager A, Konermann A (2022). Role of chaperone-assisted selective autophagy (CASA) in mechanical stress protection of periodontal ligament cells. J Orofac Orthop.

[39] Martin TG, Delligatti CE, Muntu NA, Stachowski-Doll MJ, Kirk JA (2022). Pharmacological inhibition of BAG3-HSP70 with the proposed cancer therapeutic JG-98 is toxic for cardiomyocytes. J Cell Biochem.

[40] Ji C, Tang M, Zeidler C, Hohfeld J, Johnson GV (2019). BAG3 and SYNPO (synaptopodin) facilitate phospho-MAPT/Tau degradation via autophagy in neuronal processes. Autophagy.

[41] Klimek C, Jahnke R, Wordehoff J, Kathage B, Stadel D, Behrends C, Hergovich A, Hohfeld J (2019). The Hippo network kinase STK38 contributes to protein homeostasis by inhibiting BAG3-mediated autophagy. Biochim Biophys Acta Mol Cell Res.

[42] Ma S, Meng Z, Chen R, Guan KL (2019). The Hippo pathway: biology and pathophysiology. Annu Rev Biochem.

[43] Russell JO, Camargo FD (2022). Hippo signalling in the liver: role in development, regeneration and disease. Nat Rev Gastroenterol Hepatol.

[44] Calses PC, Crawford JJ, Lill JR, Dey A (2019). Hippo pathway in cancer: aberrant regulation and therapeutic opportunities. Trends Cancer.

[45] Liu J, Ye L, Li Q, Wu X, Wang B, Ouyang Y, Yuan Z, Li J, Lin C (2018). Synaptopodin-2 suppresses metastasis of triple-negative breast cancer via inhibition of YAP/TAZ activity. J Pathol.

[46] Gan L, Camarena V, Mustafi S, Wang G (2019). Vitamin C inhibits triple-negative breast cancer metastasis by affecting the expression of YAP1 and synaptopodin 2. Nutrients.

[47] Ouyang C, Xie Y, Fu Q, Xu G (2021). SYNPO2 suppresses hypoxia-induced proliferation and migration of colorectal cancer cells by regulating YAP-KLF5 axis. Tissue Cell.

[48] Kai F, Tanner K, King C, Duncan R (2012). Myopodin isoforms alter the chemokinetic response of PC3 cells in response to different migration stimuli via differential effects on Rho-ROCK signaling pathways. Carcinogenesis.

[49] Yu L, Wei J, Liu P (2022). Attacking the PI3K/Akt/mTOR signaling pathway for targeted therapeutic treatment in human cancer. Semin Cancer Biol.

[50] Jiang T, Wang H, Liu L, Song H, Zhang Y, Wang J, Liu L, Xu T, Fan R, Xu Y (2021). CircIL4R activates the PI3K/AKT signaling pathway via the miR-761/TRIM29/PHLPP1 axis and promotes proliferation and metastasis in colorectal cancer. Mol Cancer.

[51] Xia E, Zhou X, Bhandari A, Zhang X, Wang O (2018). Synaptopodin-2 plays an important role in the metastasis of breast cancer via PI3K/Akt/mTOR pathway. Cancer Manag Res.

[52] Shiovitz S, Korde LA (2015). Genetics of breast cancer: a topic in evolution. Ann Oncol.

[53] Lee E, Jones V, Topkas E, Harraway J (2021). Reduced sensitivity for EGFR T790M mutations using the Idylla EGFR mutation test. J Clin Pathol.

[54] Kuchenbaecker KB, Ramus SJ, Tyrer J, Lee A, Shen HC, Beesley J, Lawrenson K, Mcguffog L, Healey S, Lee JM (2015). Identification of six new susceptibility loci for invasive epithelial ovarian cancer. Nat Genet.

[55] Kim JH, Cheong HS, Park JS, Jang AS, Uh ST, Kim YH, Kim MK, Choi IS, Cho SH, Choi BW (2013). A genome-wide association study of total serum and mite-specific IgEs in asthma patients. PLoS ONE.

[56] Tian L, Long F, Hao Y, Li B, Li Y, Tang Y, Li J, Zhao Q, Chen J, Liu M (2022). A cancer associated fibroblasts-related six-gene panel for anti-PD-1 therapy in melanoma driven by weighted correlation network analysis and supervised machine learning. Front Med.

[57] Zheng J, Wang L, Cheng Z, Pei Z, Zhang Z, Li Z, Zhang X, Yan D, Xia Q, Feng Y (2018). Molecular changes of lung malignancy in HIV infection. Sci Rep.

[58] Esteban S, Moya P, Fernandez-Suarez A, Vidaurreta M, Gonzalez-Peramato P, Sanchez-Carbayo M (2012). Diagnostic and prognostic utility of methylation and protein expression patterns of myopodin in colon cancer. Tumour Biol.

[59] Cebrian V, Alvarez M, Aleman A, Palou J, Bellmunt J, Gonzalez-Peramato P, Cordon-Cardo C, Garcia J, Piulats JM, Sanchez-Carbayo M (2008). Discovery of myopodin methylation in bladder cancer. J Pathol.

[60] Alvarez-Mugica M, Cebrian V, Fernandez-Gomez JM, Fresno F, Escaf S, Sanchez-Carbayo M (2010). Myopodin methylation is associated with clinical outcome in patients with T1G3 bladder cancer. J Urol.

[61] Sirover MA (2018). Pleiotropic effects of moonlighting glyceraldehyde-3-phosphate dehydrogenase (GAPDH) in cancer progression, invasiveness, and metastases. Cancer Metastasis Rev.

[62] Dey A, Varelas X, Guan KL (2020). Targeting the Hippo pathway in cancer, fibrosis, wound healing and regenerative medicine. Nat Rev Drug Discov.

[63] He Y, Sun MM, Zhang GG, Yang J, Chen KS, Xu WW, Li B (2021). Targeting PI3K/Akt signal transduction for cancer therapy. Signal Transduct Target Ther.

[64] Kirtonia A, Gala K, Fernandes SG, Pandya G, Pandey AK, Sethi G, Khattar E, Garg M (2021). Repurposing of drugs: an attractive pharmacological strategy for cancer therapeutics. Semin Cancer Biol.

[65] Yu YP, Tseng GC, Luo JH (2006). Inactivation of myopodin expression associated with prostate cancer relapse. Urology.

[66] De Ganck A, De Corte V, Bruyneel E, Bracke M, Vandekerckhove J, Gettemans J (2009). Down-regulation of myopodin expression reduces invasion and motility of PC-3 prostate cancer cells. Int J Oncol.

[67] Jing L, Liu L, Yu YP, Dhir R, Acquafondada M, Landsittel D, Cieply K, Wells A, Luo JH (2004). Expression of myopodin induces suppression of tumor growth and metastasis. Am J Pathol.

